# Marginal cord insertion in the first trimester is associated with furcate cord insertion

**DOI:** 10.1186/s12884-024-06562-4

**Published:** 2024-06-15

**Authors:** Zhuan Yu, Yu-Zhou Liu, Zheng Zhang, Bao-Ding Chen, Xin Zhang

**Affiliations:** 1 Department of Medical Ultrasonics, Affiliated Hospital of Jiangsu University, Zhenjiang, 212001 China; 2 Department of Obstetrics, Affiliated Hospital of Jiangsu University, Zhenjiang, 212001 China

**Keywords:** Marginal cord insertion, Furcate cord insertion, Prenatal diagnosis, Ultrasound

## Abstract

**Objectives:**

To evaluate the potential connections between marginal cord insertion during the first trimester and furcate cord insertion later in pregnancy.

**Methods:**

This is a prospective study of screening data on the cord insertion site in 3178 singleton pregnancies. The cord insertion site was examined in two stages. The first stage was screening for the cord insertion site between 10–13 weeks of gestation, the purpose is to determine the category of umbilical cord insertion. The second stage, performed at 22–28 weeks of gestation, was to follow up on the relationship between the cord insertion site and the placenta and to identify any changes in the category of umbilical cord insertion. This was performed to diagnose or exclude furcate cord insertion by identifying whether the umbilical cord trunk separated or branched before it reached the placenta. Factors influencing progression to furcate cord insertion and perinatal complications were assessed.

**Results:**

Fourteen cases (0.44%) with progression to furcate cord insertion, all of which showed marginal cord insertion on ultrasound in the first trimester (*p* < 0.001). without progression to furcate cord insertion, there were no changes in the category of umbilical cord insertion in 3050 cases (96.40%) compared to the early pregnancy. 114 cases (3.60%) with changes in the category of umbilical cord insertion that was not consistent with furcate cord insertion. A total of 14 cases progressed to furcate cord insertion, all showed the cord insertion site were in close proximity, and 11 (78.57%) cases showed a low insertion site (*p* < 0.001). Regarding the choice of mode of delivery, elective caesarean delivery was done in 8/14 (57.14%). The incidences of spontaneous vaginal delivery were 5/14 (35.71%) (*p* < 0.001). One (7.14%) case of progression to furcate cord insertion due to haematoma at the root of the umbilical cord ended with an emergency caesarean section. In terms of perinatal complications, marginal cord insertion that progressed to furcate cord insertion had higher incidences of SGA infants, abnormal placental morphology, retention of the placenta, and cord-related adverse pregnancy outcomes than not progressed to furcate cord insertion (*p* < 0.05).

**Conclusions:**

Marginal cord insertion in the first trimester has the potential to progress to furcate cord insertion. We suggest that ultrasound-diagnosed marginal cord insertion in the first trimester should be watched carefully in the second trimester, which is clinically useful to accurately determine the category of cord insertion and to improve the rate of prenatal diagnosis of furcate cord insertion.

## Background

Marginal cord insertion and velamentous cord insertion are the two traditional categories of abnormal cord insertion. In recent years, as the state of the art has advanced, this concept has been further expanded, some scholars proposing a new classification of umbilical cord insertion anomalies: furcate cord insertion. In the definition of furcate cord insertion, the umbilical cord trunk separates or branches before reaching the placenta rather than entering directly, and the umbilical vessel branches that are not covered by Wharton’s jelly are centred**,** eccentric, or marginally inserted into the placenta or into nearby amniotic membranes before entering the placenta [1]. The branches of the umbilical vessels are more vulnerable to twisting, dilatation, aneurysm, rupture, and thrombosis as a result of the absence of Wharton’s jelly and the separation of the umbilical vessels, which are major causes of unexplained prenatal haemorrhagic amniotic fluid and infant death [2]. A precise diagnosis of furcate cord insertion during the prenatal period is essential for the clinical selection of delivery technique, decreasing emergency caesarean deliveries, and reducing neonatal death.

Marginal cord insertion is defined as a cord insertion site within 20 mm of the placental edge [3]. Most studies have determined that marginal cord insertion has little effect on the mother or infant and does not require follow-up since the umbilical cord root is covered and protected by Wharton’s jelly, and the umbilical vessel branches in tight spirals are inserted directly into the placental parenchyma [3–6]. It has been suggested recently that the connection between the cord insertion site and the placenta changes as the pregnancy continues [7]. Numerous studies have demonstrated that lower uterine segment development and placental atrophy cause velamentous cord insertion to progress to vasa previa [8–11]. Additionally, it has been discovered that placental atrophy can cause marginal cord insertion to develop into velamentous cord insertion [12, 13]. This suggests that there are inconsistencies in the mode of umbilical cord insertion throughout pregnancy and that some of them change as gestational age increases. However, there have been no observed cases of marginal cord insertion developing into furcate cord insertion during pregnancy. Further research in this area is required.

Our objectives were to evaluate the possible association of marginal cord insertion detected in the first trimester with furcate cord insertion in the second trimester and to determine the influencing factors and perinatal complications the progression of marginal cord insertion to furcate cord insertion.

## Methods

This prospective study included 3,831 pregnant women recruited between April 2020 and January 2023 at the Department of Medical Ultrasonics, Affiliated Hospital of Jiangsu University. We excluded pregnant women who met the following criteria: (1) no birth record at the study hospital; (2) non-singleton pregnancies; (3) no follow-up data; (4) failure to specify the cord insertion site. The study design was approved by the Ethics Committee of the Affiliated Hospital of Jiangsu University (approval no.: SWYXLL20200429-8). Study participation was voluntary. All enrolled patients provided written informed consent.

The cord insertion site was examined in two stages. The first stage was screening for the cord insertion site between 10–13 weeks of gestation. Each participant’s site of insertion was identified using two-dimensional greyscale combined with colour or energy Doppler in the sagittal and cross-sectional planes. The relationship of the cord insertion site to the chorion villosum and the placenta was evaluated and recorded as marginal cord insertion, velamentous cord insertion, normal cord insertion and furcate cord insertion. Furcate cord insertion was observed and the types of insertion were recorded: the central, eccentric and marginal insertions. Marginal cord insertion was observed and the distance between the cord insertion site and the placental edge was then recorded, with distances of ≤ 5 mm marked as close proximity and distances of > 5 mm marked as long proximity. At the same time, the cord insertion site was classified subjectively as being in the upper, middle or lower third of the uterus, with visualization of the cord insertion site near the lower third of the uterus defined as low cord insertion and insertion near the upper and middle uterus considered normal insertion. The position, morphology, and internal echo of the placenta were all carefully observed and recorded.

**Fig. 1:**
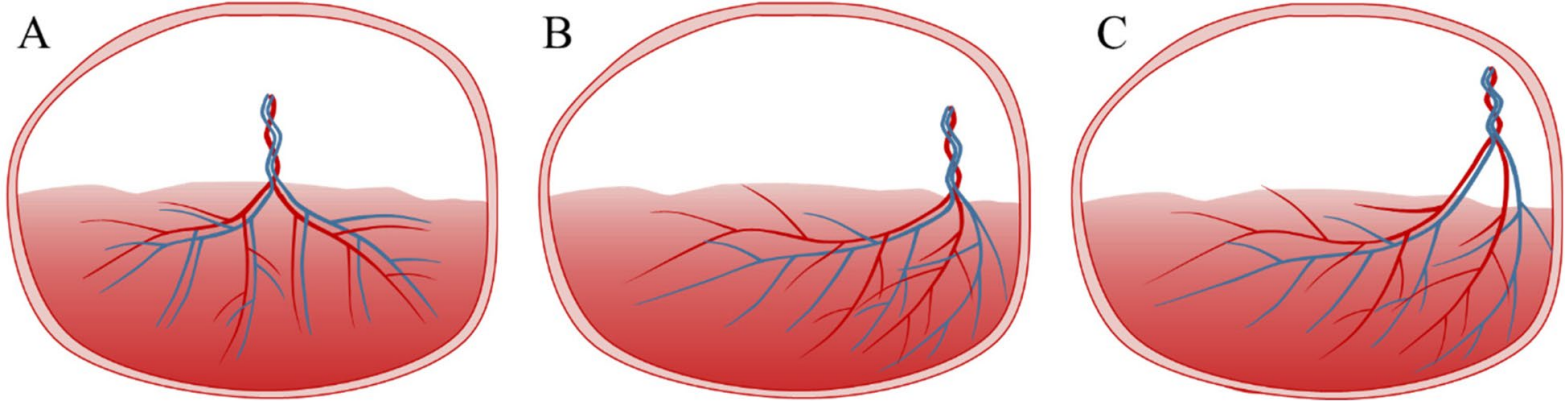
Variations in the mode of marginal cord insertion. **A** migration to normal cord insertion, **B** persistent marginal cord insertion, **C** progression to furcate cord insertion

**Fig. 2:**
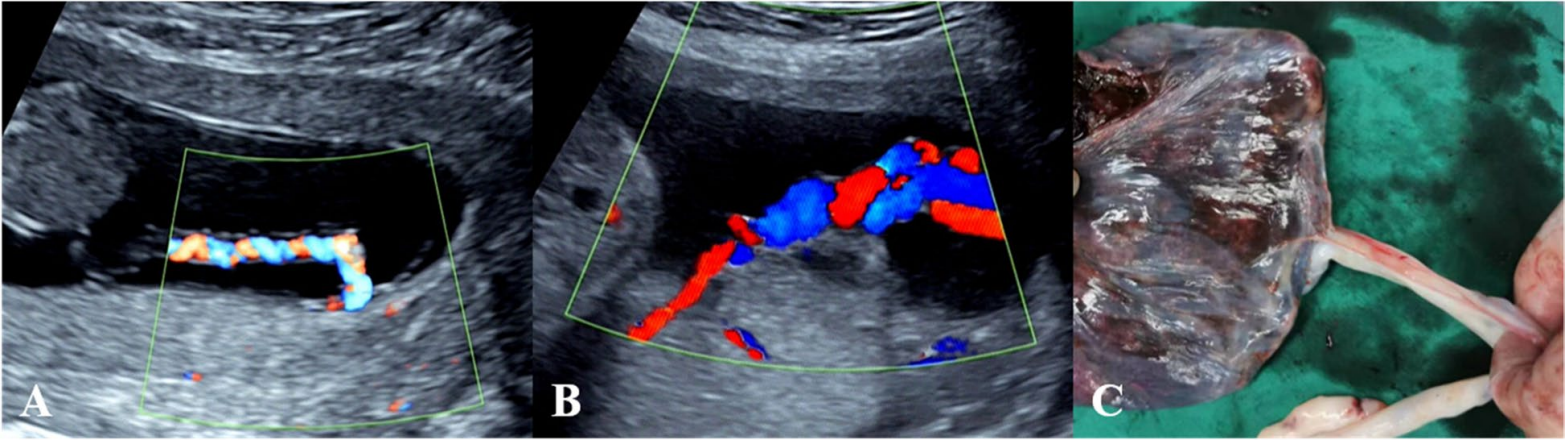
Ultrasound sonograms and gross specimen with persistent marginal cord insertion showed umbilical vessel branches in tight spirals inserted directly into the placental parenchyma. **a** Energy Doppler of marginal cord insertion in the first trimester; **b** Colour Doppler of marginal cord insertion in the second trimester; **c** Gross specimen with marginal cord insertion showed Wharton’s jelly wrapping around the root of the cord

**Fig. 3:**
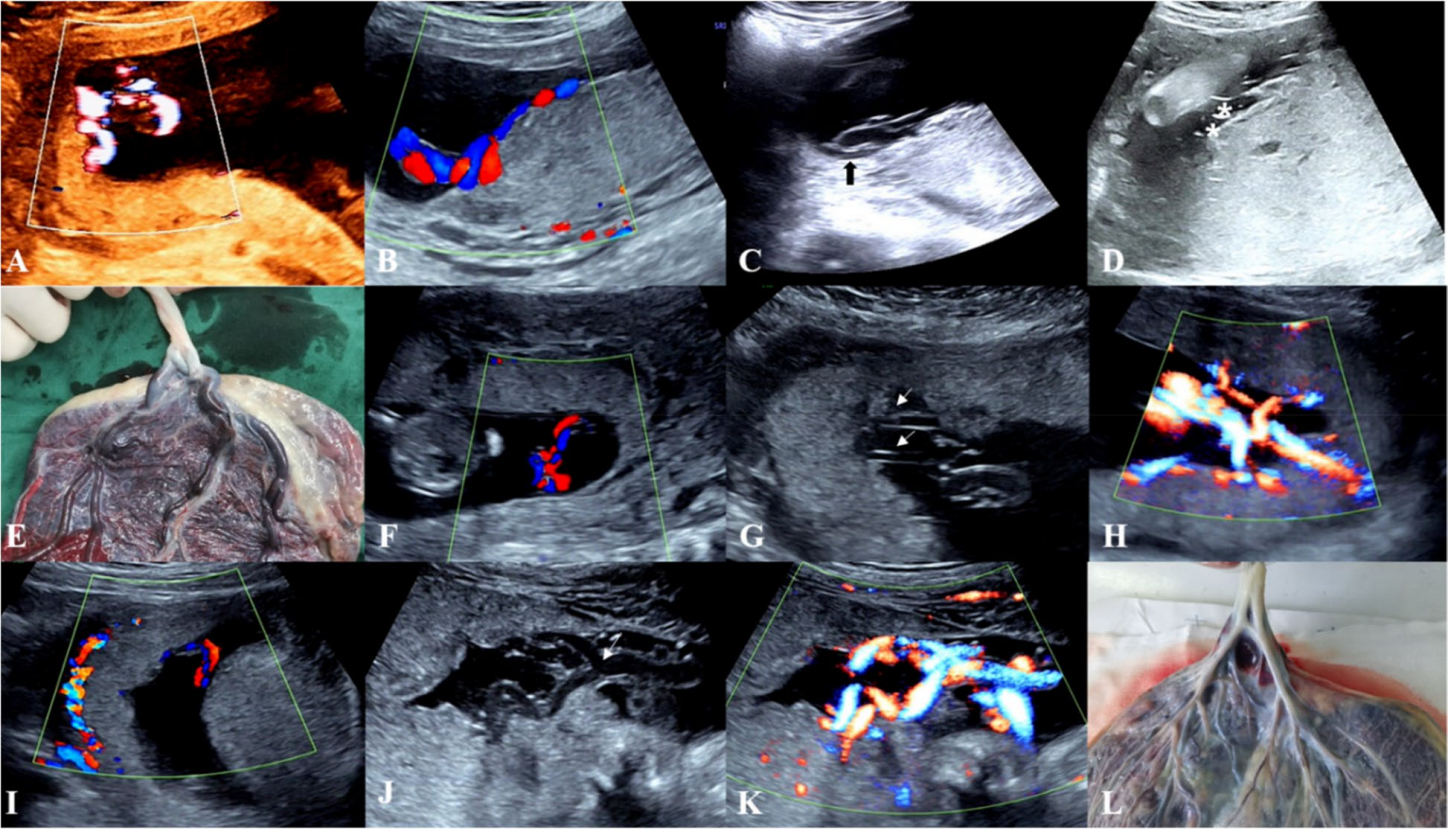
Ultrasound sonograms and gross specimen with marginal cord insertion progression to furcate cord insertion. **a** Energy Doppler of furcate cord insertion in the first trimester; **b**, **f**, **i** Colour Doppler of marginal cord insertion showed umbilical vessel branches in tight spirals directly entering the placental parenchyma. **c**, **d**, **g**, **j** 2D greyscale diagram of marginal cord insertion progression to furcate cord insertion showed small and separated (Figure **d** asterisks, Figure **g** arrow) or branched (Figure **j** arrow) umbilical vessels before entering the placenta parenchyma, with partial umbilical vessel branches travelling through the amniotic membranes (Figure **c** black arrow); **h**, **k** Energy Doppler of furcate cord insertion in the second trimester; **e**, **l** Gross specimens with furcate cord insertion showed separation or branching of the umbilical cord trunk before entering the placenta parenchyma, and the umbilical vessel branches were not wrapped by Wharton’s jelly, confirming furcate cord insertion

The second stage, performed at 22–28 weeks of gestation, was to follow up on the relationship between the cord insertion site and the placenta and to identify any changes in the category of umbilical cord insertion. This was performed to diagnose or exclude furcate cord insertion by identifying whether the umbilical cord trunk separated or branched before it reached the placenta, to record the path of the umbilical vessel branches and to determine their relationship with the placenta and amniotic membranes. We recorded the following findings: changes in the category of umbilical cord insertion compared with the early pregnancy and the umbilical cord trunk divided or separated into multiple vessels before reaching the placenta and then entered the placental parenchyma or the nearby amniotic membranes were considered clear progression to furcate cord insertion. The following two conditions were considered not progression to furcate cord insertion: no changes in the category of umbilical cord insertion compared to the early pregnancy; changes in the category of umbilical cord insertion, but not consistent with furcate cord insertion (Fig. 1 ). The examinations were performed by 10 different ultrasonographers who had more than 5 years of experience in prenatal examination. The examiners were trained for this study and discussed the diagnostic and observational components of cord insertion before the study. All checks were performed using a Voluson E10 (GE Healthcare Ultrasound, Milwaukee, Wisconsin, USA) and a Voluson 730 (GE Medical Systems Kretztechnik GmbH & Co. OHG) equipped with a 3.5–5.0 MHz probe. Abnormalities of the placenta and umbilical cord were pathologically identified by obstetricians after delivery (Figs. 2 and 3). From the hospital’s medical record, we also extracted age, obstetric history, mode of conception, mode of delivery, and various maternal and infant complications discovered in the follow-up ultrasound examination or at delivery.

Obstetric history included prior miscarriage, prior caesarean section, and prior dilatation and curettage. Mode of delivery included spontaneous vaginal delivery, emergency caesarean delivery, and elective caesarean delivery. Various maternal and infant complications identified in the third trimester or at delivery included SGA infant, preterm labour, abnormal placental morphology, retention of the placenta, partial umbilical vessel branching along the amniotic membranes, cord twisting, varices, cord root haematoma, and umbilical artery atresia. SGA was defined as birth weight below the 10th percentile for gestational age. Preterm labour was defined as labour occurring from 28 weeks of gestation but less than 37 weeks. Abnormal placental morphology was included succenturiate placenta, bilobed or multilobed placenta. Umbilical artery atresia was defined as a previous ultrasound suggestive of two umbilical arteries with a single umbilical artery on this ultrasound.

Statistical analyses were performed using SPSS 22.0 software. The univariate analyses were analysis of variance for quantitative variables and Fisher’s exact test or the chi-square test for qualitative variables. Statistical significance was defined as *P* < 0.05.

## Results

**Fig. 4:**
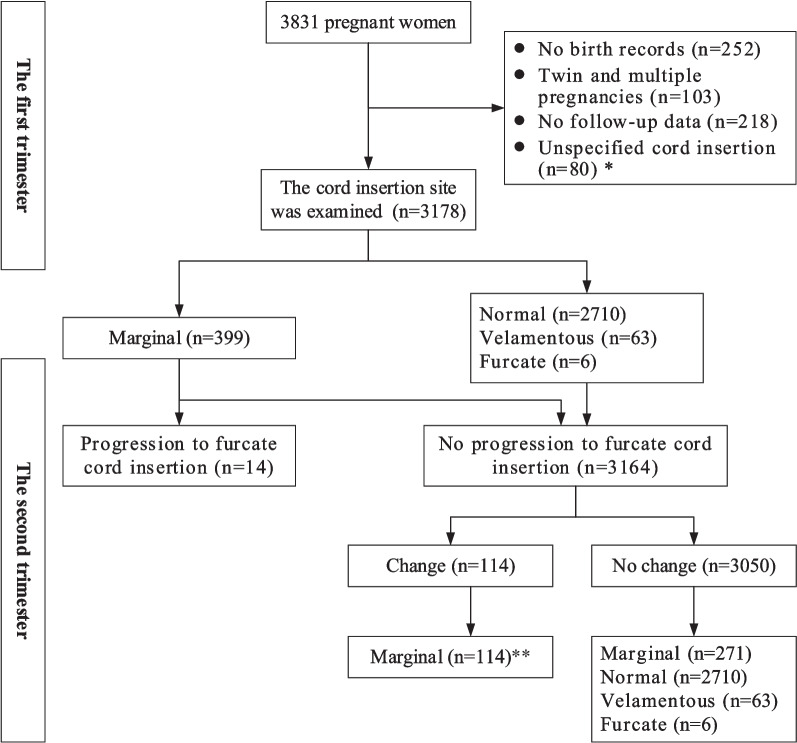
Flowchart of the study population. *Of the 43 cases in which the cord insertion site could not be specified during the first trimester, one case was confirmed to velamentous cord insertion, 38 cases were confirmed to marginal cord insertion at delivery, 4 cases were confirmed to furcate cord insertion, the site of the latter was central or eccentric insertions. Of the 37 marginal cord insertions during the first trimester in which the cord insertion site could not be specified during the second trimester, 3 cases were confirmed to velamentous cord insertion, 34 cases were confirmed to marginal cord insertion at delivery. **Compared to early pregnancy, 114 cases with changes in the category of umbilical cord insertion were not consistent with furcate cord insertion, all of which were marginal cord insertions during the first trimester migrating to normal cord insertion

Of the 3,831 participants in the prospective study, we excluded 355 pregnant women, including 252 without a birth record at the study hospital, and 103 with twin and multiple pregnancies. 3476 pregnant women who were scheduled to deliver at the hospital were enrolled to assess the cord insertion site in the first trimester. 43 pregnancies were excluded in which the cord insertion site could not be specified during the first trimester, meanwhile, the cord insertion site was classified sonographically into cases of 2710 normal cord insertions, 458 marginal cord insertions, 6 furcate cord insertions, and 63 velamentous cord insertion. In the second trimester, we excluded 37 pregnancies in which we could not specify the umbilical cord insertion and 218 pregnancies in which follow-up data were missing. 14 cases (0.44%) with progression to furcate cord insertion, all of which showed marginal cord insertion on ultrasound in the first trimester. 3164 cases (99.56%) without progression to furcate cord insertion, there were no changes in the category of umbilical cord insertion compared to the early pregnancy in 3050 cases (96.40%), of which 2710 cases (88.85%) were normal cord insertion, 63 cases (2.07%) were velamentous cord insertion, 271 cases (8.89%) were marginal cord insertion, and 6 cases (0.20%) were furcate cord insertion, the site of the latter was central or eccentric insertions. Compared to early pregnancy, 114 cases (3.60%) with changes in the category of umbilical cord insertion were not consistent with furcate cord insertion, all of which were marginal cord insertions during the first trimester migrating to normal cord insertion (Table 1). Of the 43 cases in which the cord insertion site could not be specified during the first trimester, one case was confirmed to velamentous cord insertion, 38 cases were confirmed to marginal cord insertion at delivery, 4 cases were confirmed to furcate cord insertion, the site of the latter was central or eccentric insertions. Of the 37 marginal cord insertions during the first trimester in which the cord insertion site could not be specified during the second trimester, 3 cases were confirmed to velamentous cord insertion, 34 cases were confirmed to marginal cord insertion at delivery. Figure 4 shows the flowchart of the study population.

**Table 1:** Relationship between categories of umbilical cord insertion in the first trimester and progression to furcate cord insertion

Categories of cord insertion	Progression to furcate cord insertion (*n* = 14)	No progression to furcate cord insertion (*n* = 3164)	*P*-value
Normal cord insertion	0	2710 (85.65)	< 0.0001
Marginal cord insertion	14 (100)	385 (12.17)	< 0.0001
Velamentous cord insertion	0	63 (1.99)	1.000
Furcate cord insertion	0	6	1.000

A total of 14 cases progressed to furcate cord insertion, all showed the cord insertion site were in close proximity, and 11 (78.57%) cases showed a low insertion site. Of 385 marginal cord insertion that did not progress to furcate cord insertion, 87 (22.60%) showed the cord insertion site were in close proximity, and 71 (18.44%) showed a low insertion site. All these differences were statistically significant (*p* < 0.001) (Table 2).

**Table 2:** Age, pregnancy characteristics, and ultrasound observables of the marginal cord insertion in the first trimester according to pogression to furcate cord insertion

Characteristics	Progression to furcate cord insertion (*n* = 14)	No progression to furcate cord insertion (*n* = 385)	*P*-value
Age	30.29 ± 2.12	29.22 ± 3.82	0.299
Prior miscarriage	3 (21.43)	12 (3.12)	0.012
Prior cesarean section	2 (14.29)	13 (3.38)	0.092
Prior dilatation and curettage	3 (21.43)	12 (3.12)	0.012
IVF/IUI	1 (7.14)	2 (0.52)	0.102
Placental attachment position			
Anterior wall	7 (50)	100 (25.97)	0.063
Posterior wall	2 (14.29)	108 (28.05)	0.367
Sidewall	2 (14.29)	74 (19.22)	1.000
Mixed wall	3 (21.43)	103 (26.75)	0.768
The distance between the cord insertion site and the placental edge			< 0.0001
Close proximity	14 (100)	87 (22.60)	
Long proximity	0 (0)	298 (77.40)	
The relationship between the cord insertion site and the uterus			< 0.0001
Low insertion site	11 (78.57)	71 (18.44)	
Normal insertion site	3 (21.43)	314 (81.56)	

Data are expressed as mean ± SD or n (%)

*Abbreviations*: *IUI* intrauterine insemination, *IVF* in vitro fertilization

Regarding the choice of mode of delivery, elective caesarean delivery was done in 8/14 (57.14%) and 29/385 (7.53%) of the marginal cord insertion progressed to furcate cord insertion and not progressed to furcate cord insertion, respectively. The respective incidences of spontaneous vaginal delivery were 5/14 (35.71%) and 355/385 (92.21%) (*p* < 0.001). One (7.14%) case of progression to furcate cord insertion due to haematoma at the root of the umbilical cord and one (0.26%) case of no progression to furcate cord insertion due to atresia of the umbilical artery ended with emergency caesarean section. In terms of perinatal complications, marginal cord insertion that progressed to furcate cord insertion had higher incidences of SGA infants, abnormal placental morphology, retention of the placenta, and cord-related adverse pregnancy outcomes than not progressed to furcate cord insertion (*p* < 0.05) (Table 3).

**Table 3:** Association between marginal cord insertion in the first trimester and perinatal complications according to progression to furcate cord insertion

Complication	Progression to furcate cord insertion (*n* = 14)	No progression to furcate cord insertion (*n* = 385)	*P*-value
SGA infant	3 (21.43)	13 (3.38)	0.015
Preterm labour	1 (7.14)	1 (0.26)	0.069
Mode of delivery			
Spontaneous vaginal delivery	5 (35.71)	355 (92.21)	< 0.0001
Emergency caesarean delivery	1 (7.14)	1 (0.26)	0.069
Elective caesarean delivery	8 (57.14)	29 (7.53)	< 0.0001
Abnormal placental morphology	3 (21.43)	6 (1.56)	0.003
Retention of the placenta	5 (35.71)	11 (2.86)	< 0.0001
Cord-related adverse pregnancy outcomes			
Partial umbilical vessel branching along the amniotic membranes	3 (21.43)	0 (0)	< 0.0001
Cord twist	1 (7.14)	0 (0)	0.035
Varices	1 (7.14)	0 (0)	0.035
Cord root haematoma	2 (14.29)	0 (0)	0.001
Umbilical artery atresia	0 (0)	1 (0.26)	1.000

Data are expressed as n (%)

*Abbreviation*: *SGA* small-for-gestational age

## Discussion

The umbilical cord is usually inserted into the placental tissue and is near or in the centre of the placental body. Pathological cord insertion generally includes marginal cord insertion and especially velamentous cord insertion, which is a hot topic of research on cord insertion abnormalities due to the higher perinatal mortality, but other types of cord insertion abnormalities are largely underrecognized. This study reports on a rare anomaly of umbilical cord insertion: furcate cord insertion. In our study 58.33% (14/24) cases of furcate cord insertion were progression of marginal cord insertion in the first trimester, the site of insertion was a marginal insertion, whereas in 41.67% (10/24) of cases, the site of insertion was a central or eccentric insertion. Kosian et al. [2] showed the relationship between the site of insertion and furcate cord insertion, but in their studies, 132 cases of furcate cord insertion obtained differential results with a central or eccentric insertion rate of 75% and a marginal insertion rate of 25%. This discrepancy may be primarily attributable to the fact that our study was prospective and the study population itself was patients with abnormal cord insertion, which may have increased the prevalence of furcate cord insertion. The higher incidence may also be due to the gradual advancement of knowledge about this abnormal insertion, which has increased the rate of prenatal diagnosis and consequently increased the prevalence of furcate cord insertion. Kosian et al. [2] reported a 1.0% risk of intrauterine death from furcate cord insertion, but in their studies, the incidence of intrauterine fetal death was much higher in patients with above-average long furcate cord insertion. After analyzing our data, we found that the furcate cord insertion we observed progressed from marginal cord insertion in the first trimester with a shorter distance of separated umbilical vascular branches [2], and the small sample size may be a contributing factor to the absence of intrauterine death in this cohort.

The pathogenesis of furcate cord insertion remains uncertain. Ottow B [14] described a hypothesis about its pathogenesis; for unknown reasons, the mesoderm layer between the vessels does not develop or later degenerates. In the course of pregnancy, the placenta grows, and these separated vessels can grow even further apart. However, it appears that only the early diagnosis of furcate cord insertion is explained by this. In this study, 6 cases already met the ultrasonographic diagnosis of furcate cord insertion in the first trimester. 14 cases were diagnosed by ultrasound as marginal cord insertion in the first trimester, and the umbilical cord trunk was unbranched before entering the placenta, but the category of umbilical cord insertion changed during the second trimester and progressed to furcate cord insertion. We consider this to be a possible secondary change. The part of the cord insertion may be normal at the initial stage and degeneration of the placental tissue below the location of the cord insertion occurs at a later stage, which is consistent with Fujita Y et al. [15].

We also noted that marginal cord insertion with the cord insertion site ≤ 5 mm from the placental edge in the first trimester was more likely to progress to furcate cord insertion compared with marginal cord insertion of > 5 mm. In agreement with our findings, previous investigations have also reported that a cord insertion site nearer to the placental edge is associated with a greater incidence of specific adverse pregnancy outcomes [13]. In addition, this study found a higher incidence of low insertion sites progressing to furcate cord insertion than normal insertion sites. It has been proposed that inadequate blood supply is more likely to occur in the lower uterine segment [16], marginal cord insertion in the lower uterine segment is more likely to develop into velamentous cord insertion [12], and velamentous cord insertion is more likely to create vasa previa [17]. Furthermore, this study found 3 cases of low insertion sites in the first trimester, showing that the cord insertion site was located at the edge of the placenta in the second trimester, but it was not possible to distinguish whether the cord insertion site was in the placental parenchyma or the amniotic membranes, and velamentous cord insertion was confirmed after birth. This further supports the findings of Kelley BP [12]. The above observations may indicate that placental tissue atrophy below the cord insertion site of marginal cord insertion is explained by a trophotropism mechanism [18]. The villous chorion adjacent to the cord insertion site is where the placenta develops. Due to starvation, the region of the placenta that is linked to the lower section of the uterus experiences adaptive changes, and the villous chorion atrophies into a smooth chorion, which causes the cord insertion site of marginal cord insertion to shift from its original position and eventually evolve into furcate cord insertion.

By comparing the effects of different placental attachment locations on the progression of marginal cord insertion, we found that the 14 cases of marginal cord insertion that progressed to furcate cord insertion included 7 cases of placental attachment to the anterior uterine wall, an incidence of 50%. Although the rate is twice as high in placental attachment to the anterior uterine wall group it did not reach statistical significance because of the low numbers. However, Predanic M et al. [19] consider the anterior wall of the lower uterus is weaker and receives less blood supply than the anterior wall of the middle and upper uterus, but the thickness and blood supply of the posterior wall of the lower uterus are not significantly different from the posterior wall of the middle and upper uterus; the difference in blood supply between the upper and lower segments of the sidewall of the uterus is between the anterior and posterior walls, there is a stronger tendency to change the cord insertion site in the anterior placenta.

Numerous studies [1, 2, 20] have concluded that furcate cord insertions are more vulnerable to twisting, dilatation, aneurysm, rupture, and thrombosis of the umbilical vessel branches due to the absence of Wharton’s jelly and the separation of the umbilical vessels. It is clear from the cases in this study that when compared to the other group, cases in which marginal cord insertion progressed to furcate cord insertion had a significantly higher incidence of partial umbilical vessel branching along the amniotic membranes, cord twist, varices, and cord root haematoma. Second, in terms of choice of delivery method, cases involving furcate cord insertion can be delivered by spontaneous vaginal delivery and elective caesarean delivery [21, 22]. Compared to other kinds of insertion, progression to furcate cord insertion was more likely to end in spontaneous vaginal delivery and more likely to end in elective caesarean delivery. This demonstrates good prognosis for patients with furcate cord insertion that are actively followed up on and those that adopt planned delivery. Additionally, our finding of a high incidence of placental retention with furcate cord insertion is in line with a previous study [2] that discovered a placental retention incidence of 5.1% with furcate cord insertion after analysing the characteristics of 132 cases of furcate cord insertion.

Pregnant women who progressed to furcate cord insertion were much fewer than the other groups, which may represent bias in our results, and the sample size needs to be further expanded. The second limitation of this study is that as in other studies on cord insertion sites, the diagnosis of marginal cord insertion during the first trimester was based on ultrasonography and not confirmed by placental pathology. We were unable to ascertain the category of umbilical cord insertion. Despite the possibility of misdiagnosis, patients are advised of the risk of progression to furcate cord insertion based on ultrasound diagnosis. The third limitation of our study is the use of only transabdominal probes to classify the types of umbilical cord insertion, resulting in missing a portion of cases. Another limitation is that this study only applies to singleton pregnancies. In addition, our study has not observed cases in which normal cord insertion, velamentous cord insertion progressed to furcate cord insertion, and further studies are needed to confirm the existence of this phenomenon.

In conclusion, marginal cord insertion detected by ultrasound in the first trimester should alert clinicians to the possibility of progression to furcate cord insertion, which should be watched for in the second trimester. The changes in the cord insertion site were comprehensively evaluated according to the distance of the cord insertion site from the placenta edge, and the relationship between the cord insertion site and the uterus, accurately determining the type of cord insertion. Any separation or branching of the umbilical vessels before the insertion of the placenta or adjacent to the amniotic membranes was observed to improve the prenatal diagnosis of furcate cord insertion, and the umbilical vessel branches were closely followed up for twisting, dilatation, aneurysm, and rupture to minimize the occurrence of perinatal adverse pregnancy.

## Data Availability

The datasets analyzed during the current study are available from the corresponding author upon reasonable request.

## Abbreviations

SGA: Small-for-gestational age
IVF: In-vitro fertilization
IUI: Intrauterine insemination
